# Applications of Single-Cell Omics in Tumor Immunology

**DOI:** 10.3389/fimmu.2021.697412

**Published:** 2021-06-09

**Authors:** Junwei Liu, Saisi Qu, Tongtong Zhang, Yufei Gao, Hongyu Shi, Kaichen Song, Wei Chen, Weiwei Yin

**Affiliations:** ^1^ Department of Cardiology of the Second Affiliated Hospital, Zhejiang University School of Medicine, Key Laboratory for Biomedical Engineering of the Ministry of Education, College of Biomedical Engineering and Instrument Science, Zhejiang University, Hangzhou, China; ^2^ Department of Hepatobiliary and Pancreatic Surgery, The Center for Integrated Oncology and Precision Medicine, Affiliated Hangzhou First People’s Hospital, Zhejiang University School of Medicine, Hangzhou, China; ^3^ School of Mechanical Engineering, Zhejiang University, Hangzhou, China; ^4^ Department of Biological Testing, Zhejiang Puluoting Health Technology Co., Ltd., Hangzhou, China; ^5^ Department of Thoracic Surgery, Sir Run Run Shaw Hospital, Zhejiang University, Hangzhou, China

**Keywords:** single-cell omics, immunotherapy, cancer, TCR (T cell receptor), biomarkers

## Abstract

The tumor microenvironment (TME) is an ecosystem that contains various cell types, including cancer cells, immune cells, stromal cells, and many others. In the TME, cancer cells aggressively proliferate, evolve, transmigrate to the circulation system and other organs, and frequently communicate with adjacent immune cells to suppress local tumor immunity. It is essential to delineate this ecosystem’s complex cellular compositions and their dynamic intercellular interactions to understand cancer biology and tumor immunology and to benefit tumor immunotherapy. But technically, this is extremely challenging due to the high complexities of the TME. The rapid developments of single-cell techniques provide us powerful means to systemically profile the multiple omics status of the TME at a single-cell resolution, shedding light on the pathogenic mechanisms of cancers and dysfunctions of tumor immunity in an unprecedently resolution. Furthermore, more advanced techniques have been developed to simultaneously characterize multi-omics and even spatial information at the single-cell level, helping us reveal the phenotypes and functionalities of disease-specific cell populations more comprehensively. Meanwhile, the connections between single-cell data and clinical characteristics are also intensively interrogated to achieve better clinical diagnosis and prognosis. In this review, we summarize recent progress in single-cell techniques, discuss their technical advantages, limitations, and applications, particularly in tumor biology and immunology, aiming to promote the research of cancer pathogenesis, clinically relevant cancer diagnosis, prognosis, and immunotherapy design with the help of single-cell techniques.

## Introduction

The tumor microenvironment (TME) is a complex ecosystem that consists of many different cell types, including tumor cells, immune cells, and many others. All these cells are tightly inter-associated and interact with each other. The heterogeneous milieu of TME induces various progression patterns of different cancers and leads to distinct treatment responses across different patients (1). Among that, the levels of T cell infiltration, the polarization of tumor-associated macrophages (TAM) can be varied, thereby affecting the prognosis of patients differently, the expression of PD-1 and PD-L1 in TME, the mutational landscapes, and the drug responses of malignant cells can also be distinct in different patients, relating to different efficacies of immune checkpoint blockade (ICB) therapies (2–4).

The previous genomic, transcriptomic, and proteomic cancer studies have helped develop multiple mutational- or molecular-target therapies and elevate treatment responses across different patients (5). However, the clinical benefits of these target-directed therapies are still limited. Only a small subset of patients is treatable, leading to emergent demand of using more precise methods to dissect characteristics of individual patients for developing better cancer treatments especially personalized tumor immunotherapy.

In this review, we introduce the state-of-art technological advances of single-cell omics and discuss corresponding computational methods for single-cell data analysis and their applications in cancer research. All of these further inspire and guide the design of and applications of single-cell techniques in basic and translational clinical cancer research.

## The Development of Single-Cell Technologies

**Figure 1:**
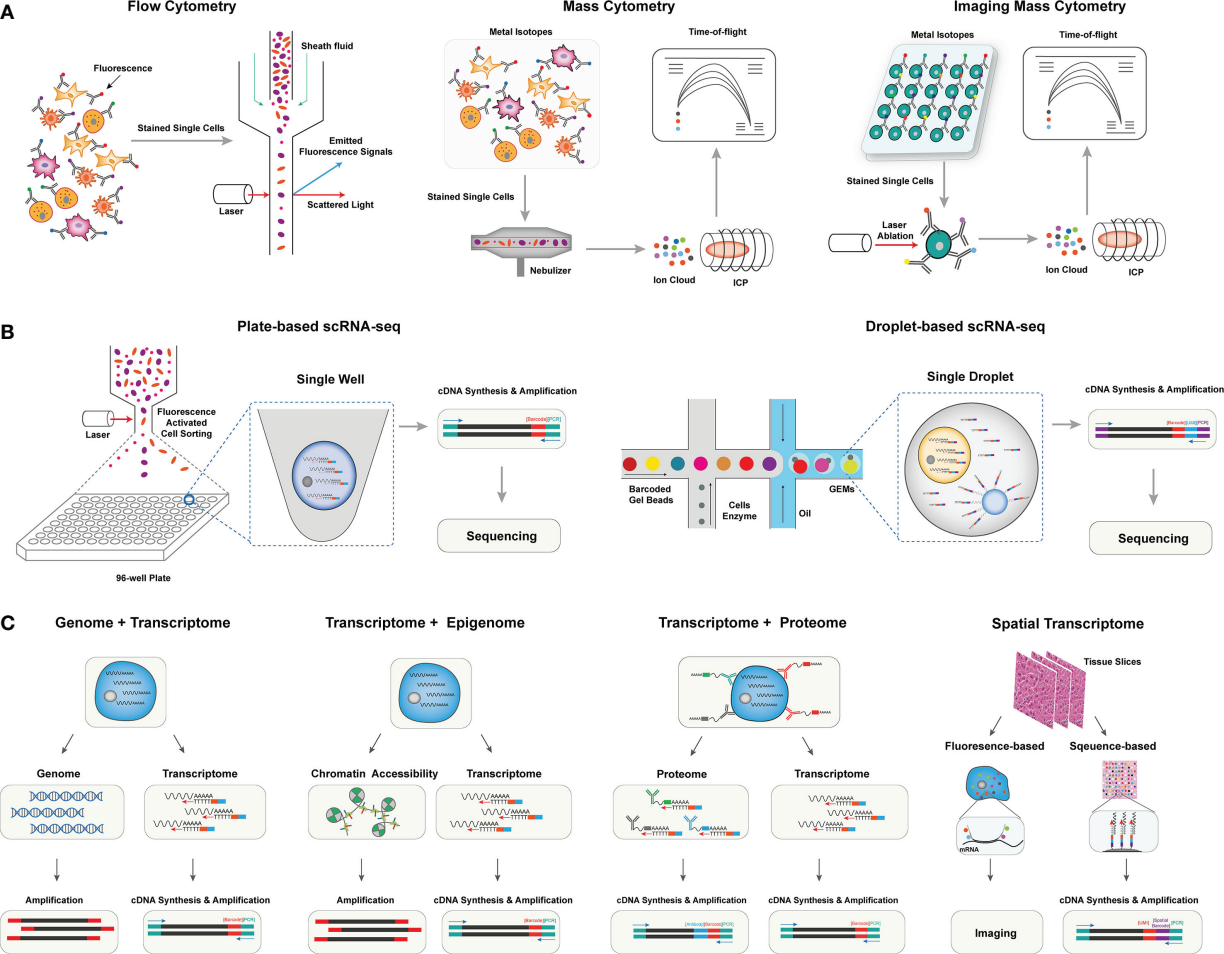
The overview of single-cell omics techniques. **(A)** The overview of single-cell cytometry systems, including flow cytometry with fluorescence-labeled antibodies for single isolated cells (left), mass cytometry with metal isotope-conjugated antibodies isolated single cells (middle), and imaging mass cytometry with metal isotope-conjugated antibodies labeled on tissues (right). **(B)** The overview of two canonical scRNA-seq platforms, including plate-based scRNA-seq methods with sorted cells barcoded within each well (left), and droplet-based scRNA-seq method, single cells were barcoded within individual droplets (right). **(C)** The overview of single-cell multi-omics techniques, including library preparing for genomic, epigenomic, proteomic, and spatial indexing with transcriptomic of single cells simultaneously.

The development of methods in single-cell isolation, indexing, and sequencing allows in-depth profiling of the tumor milieu from different cellular scales with extremely high dimensions (6). Based on single omics, methods for integrated omics have also been developed for simultaneous detection of different omics, including genomic, transcriptomic, proteomic, and spatial information of single cells (7). Despite multiple challenges that remain to be addressed, these methods have been very powerful in uncovering the cellular basis of the heterogeneous tumor microenvironment and greatly expanded our understanding of cancers and tumor immunology in many aspects. In this session, we introduce the technical details of single-cell methods applied in understanding the tumor microenvironment (**Figure 1**).

### Mass Cytometry and Imaging Mass Cytometry

Flow cytometry, as a widely used single-cell labeling and sorting technique, has facilitated the understanding of cellular composition and diversity in various tissues (8), but its spectral overlap between nearby channels limits the number of detected markers and unable to unveil many functionally important cell subsets (9). To overcome this problem, mass cytometry, also named cytometry by time of flight mass spectrometry (CyTOF), was developed with a more specific channel signal (10, 11). CyTOF uses rare element isotopes to replace the commonly used fluorochrome to conjugate monoclonal antibodies (mAbs) in flow cytometry. These isotopes usually do not exist in cells, and the purity of rare element isotopes and their accurate detection by mass spectrometry significantly increase the detectable dimension of a single cell to over 100 markers theoretically, and due to the technical limits of isotope labeling onto mAbs, the marker number on single cells to date can only reach 45 (10–12). Meanwhile, CyTOF has already demonstrated its power and accuracy over flow cytometry in cell profiling when applied to analyze fresh and frozen PBMC or tumor tissues at the single-cell level (13). Unfortunately, CyTOF cannot be used for cell sorting, and its throughput is 25~50 times lower than flow cytometry due to extra time expense for isotope quantification (8).

Beyond profiling the homogenously stained single cells isolated from tissue samples, imaging CyTOF was developed to profile the cells’ spatial information in the target tissue (14). Similar to the multicolor immunofluorescence staining, imaging CyTOF can simultaneously detect over 30 types of rare element isotopes conjugated on antibodies to stain tissue sections. A high-resolution laser is used to ablate the target tissue section point by point, and the ionized elements were streamed into the ICP-MS for isotope measurement. Finally, a high-dimensional tissue imaging is reconstructed by integrating the subcellular spatial information of each point on the tissue sample (14).

Moreover, CyTOF can also be used in the quantification of epigenetic modification (e.g., phosphorylation, histone modification) (15), transcripts (16), and antigen-specific T cells (17) at the single-cell level by designated mAbs that target chromatin marks, ligation assay for RNA, and multiplexed peptide-major-histocompatibility-complex (pMHC)-tetramer staining for antigen-specific T cells, respectively, allowing an integrated inspection of cellular functionality in a multi-omics manner.

### Single-Cell RNA Sequencing

Methods for profiling single-cell transcriptome have been developed and rapidly evolved to overcome limited markers detected on individual cells by CyTOF, improve the single-cell resolution of traditional bulk RNA sequencing (RNA-seq), and identify rare cell populations and their functional dynamics at the transcriptomic level (18). The first published single-cell RNA sequencing (scRNA-seq) method successfully detected 5,270 more genes in one blastomere compared to the microarray assay using hundreds of blastomeres, allowing the precise whole-transcriptome characterization at a single-cell level (19). And integrating the ‘cell-specific barcodes’ into the synthesized cDNA sequences (20, 21), the throughput of scRNA-seq improved from a few hundreds of cells to thousands of cells.

Multiple scRNA-seq or sc-nucleus RNA-seq protocols were developed to enhance the scale, the sensitivity, or the accuracy of single-cell transcriptome quantification (22). These methods can be categorized into plate-based or microfluidic-based platforms. For the plate-based platform, the representative method is Smart-seq, which is currently upgraded into a third-generation, Smart-seq3 (23). In Smart-seq3, a 5’ unique molecular identifier (UMI) is integrated into the full-length cDNA for counting transcripts, achieving the precise quantification of transcript isoforms. Other plate-based platforms, such as cell expression by linear amplification and sequencing (CEL-seq2) and massively parallel single-cell RNA sequencing (MARS-seq2), integrate the Fluidigm C1 system or liquid-handling robot to improve the data quality and reduce labor cost, respectively (24, 25). Applying a bead-based microfluidic system has dramatically pushed the field into a real high-throughput area for the microfluidic platform. In a single experiment, the microfluidic system can capture 3,000~10,000 droplets, each of which encapsulates a single-cell and a single-bead carrying specific DNA-barcoded primers (26–28). The transcripts in single droplets are then captured, reversely transcribed, and barcoded with cell barcodes and UMIs. These procedures well replace the single-well-based cell sorting and library construction steps in the plate-based platform and dramatically increase the detection number of single cells in each sample. Other strategies have also been used to profile the transcripts in single cells, such as the split-pool-based cell barcoding strategy (29–31) and the integration of beads with the microwell-based platform (32). Although there are still challenges in different aspects, such as cost, sequencing depth, and gene coverages, these scRNA-seq methods have enabled the profiling of single cells with more than thousands of genes per cell. The data dimension is significantly higher than the cytometry-based systems.

### Single-Cell Multi-Omics Technologies

The interconnections and relations of genome, epigenome, transcriptome, and proteome determine the function of single cells, which requires a comprehensive understanding of the biology process across multi-omics simultaneously at the single-cell level (33). In the following session, we will focus on reviewing single-cell multi-omics technologies, that could simultaneously measure at least two of different omics including genomics, transcriptomics, epigenomics, proteomics, and spatial information at the single-cell level.

Yin et al. introduced the sci-L3-RNA/DNA co-assay to simultaneously measure the genomics and transcriptomics in single cells (34). In sci-L3-RNA-/DNA co-assay, single cellular DNA and mRNA were respectively barcoded by Tn5 transposon intersection and by poly-T primer, both of them carrying barcoding sequences and UMIs. Both libraries were prepared with three-level split-pool indexing and linear amplification for downstream analysis. Another strategy is to physically separate the nucleus and cytosol of a single cell and construct the library for each component individually. Following this strategy, direct nuclear tagmentation and RNA sequencing (DNTR-seq) (35) separately obtained the whole-genome sequencing and full-length cDNA sequencing from single cells with ultra-high resolutions. Besides directly obtaining the whole genome of single cells, the cDNA sequences from mRNA could also be used to detect the mutation status of single cells (36, 37), especially for identifying tumor-specific mutations across different tumor cell populations.

Open chromatin regions are also important functional characteristics for revealing cellular genomic regulations. With the assay development for transposase-accessible chromatin using sequencing (ATAC-seq), exploring open chromatin in single cells becomes possible. ATAC-seq enables fast and precise epigenomic profiling by integrating the sequencing adaptors into the accessible chromatin by prokaryotic Tn5 enzyme (38). Combining ATAC-seq with single-cell isolation and barcoding techniques enables access to open chromatin in single cells (39). Moreover, as both the transposed chromatin fragments and the synthesized cDNA fragments of cellular transcripts can be adapted into the same cell barcoding ID, Cao et al. and Chen et al. successfully detected chromatin accessibility and transcriptome simultaneously at the single-cell level (40, 41). Simultaneous high-throughput ATAC and RNA expression with sequencing (SHARE-seq) is another method to evaluate the relationship between chromatin accessibility and gene expression in single cells and identify the priming role of chrome accessibility in transcriptomic regulation, which is helpful to infer cell differentiation (42). Meanwhile, multiple in silico algorithms, such as model-based analyses of transcriptome and regulome (MAESTRO) and Signac (43, 44), have been correspondingly developed to integrated analyze scRNA-seq and scATAC-seq data in single cells.

The protein expression can directly reflect the functionality and biological states of cells. As a result, flow cytometry and CyTOF have been broadly used in biological researches for protein expression quantification despite their limited dimensions compared to scRNA-seq. To overcome this limitation, Stoeckius et al. came up with the idea of using specifically designed DNA sequences to label and barcode the protein-specific mAbs (45). The detection number of antibody-labeled proteins is significantly increased to more than 200 (46), which is five times more than the detection number in CyTOF. CITE-seq uses a poly-A tail in the antibodies-conjugated oligonucleotides to achieve compatibility with the mRNA capturing system (45). And in the commercial platform (e.g., Feature Barcoding by 10X Genomics), the barcoding strategy is further improved so that the transcriptomic and proteomic libraries are barcoded with poly-A capture sequences and antibody-specific capture sequences separately (47). Besides, Zhang et al. used DNA-barcoded pMHC tetramers to specifically label and sequence antigen-specific T cells (48). A similar strategy was also used to remove experimental and amplification bias by staining oligo-labeled surface proteins ubiquitously expressed on cells from different samples (49).

The in-situ cellular spatial information is essential to accurately capture the biological functions of cells in their physiological context. It is particularly important to investigate the spatial information in the tumor microenvironment (TME), such as tissue-specific T cell infiltration, the spatial distribution, and interaction of cellular ligands and receptors, and the distribution of malignant cells, to improve our understanding of tumorigenesis and tumor-specific immune escape in TME (50). The spatial transcriptome methods can be mainly classified into fluorescence or sequencing-based methods, which have also been comprehensively reviewed by Asp et al. (51). Based on the technique of fluorescence in situ hybridization (FISH) (52), seqFISH+ enables visualization transcripts at local sites and can image more than 10,000 genes at subcellular resolution with upgraded optical resolution and barcoding strategy (53). Despite the high spatial resolution, the applications of fluorescence-based platforms are usually hampered by the intensive experiment procedures and the design of the transcript probe. In contrast, the application of cellular barcoding strategies in scRNA-seq enables in-situ barcoding of local cells in tissues. The most challenging for this strategy is to demultiplex the physical locations with the detected barcoding sequences. In Slide-seq and high-definition spatial transcriptomics (HDST) assays (54–56), arrayed barcoding beads are used to capture spatial whole-transcriptomes, and the resolution of the reconstructed spatial map depends on the designed bead arrays. In another microfluidic-based method (57), the tissue slide was separately barcoded by parallel microfluidic channels within different directions, and the different combinations of barcodes can recover the spatial information.

### Innovative Computational Methods for Single-Cell Analysis

Accompanied by the increased capability of generating high-dimensional and high-throughput single-cell data in one experiment, interpreting the biological functions of cells and functional alterations in disease status becomes even more challenging (58). Hie et al. summarized the typical computational workflow for single-cell RNA-seq data analysis, including data preprocessing, batch correction, clustering, and functional annotation of single cells (59). Among that, the methods for inferring cell lineage trajectories under different stimuli are broadly applied to understand cellular dynamics and interactions. Besides, with the development of single-cell multi-omics techniques, integrating multi-omics single-cell data is also computationally challenging (7).

Saelens et al. comprehensively benchmarked the performance of 45 trajectory inference methods (60), highlighting that the preset trajectory topology of computational methods can affect the inference results and that the performance of different methods can vary with different datasets. The most limitation of these methods, including Monocle3 (61), partition-based graph abstraction (PAGA) (62), and Slingshot (63), is that the cell trajectory estimation calculated by the cell-cell distance ignores the inherited cellular information. Instead, the innovative method RNA Velocity (64, 65) addresses this issue by quantifying the spliced and unspliced transcripts of single cells and connecting cells with similar transcripts splicing states. Another method, CytoTRACE, leverages the number of detected genes to reflect the developmental potential of single cells, providing a robust performance to delineate cellular trajectories (66). Furthermore, the DNA sequencing information, including T cell receptor sequencing, can also be used as cellular labels for inferring cellular dynamics and lineage tracing (67, 68).

The integrative analyses of single-cell multi-omics data consider the status of single cells from different scales of biological features and delineate cell types based on cell similarities in higher feature space, which are challenged by the different characteristics of single omic data and batch effects across multiple data samples. Ma et al. comprehensively summarized the data integration methods for analyzing single-cell multi-omics data (7). One strategy is to estimate the cellular distances within individual omics and then calculate the “weighted-nearest neighbor “ distance for integrated analysis of multiple-omics data (46). Another one exploits a modified statistic framework to identify low-dimensional variations across data modalities for data integration (69). In other methods, multi-view machine learning (70), canonical correlation (71), and deep generative model (72) have also been used for multi-omic single-cell data integration.

## Application of Single-Cell Omics in Tumor Immunology

**Figure 2:**
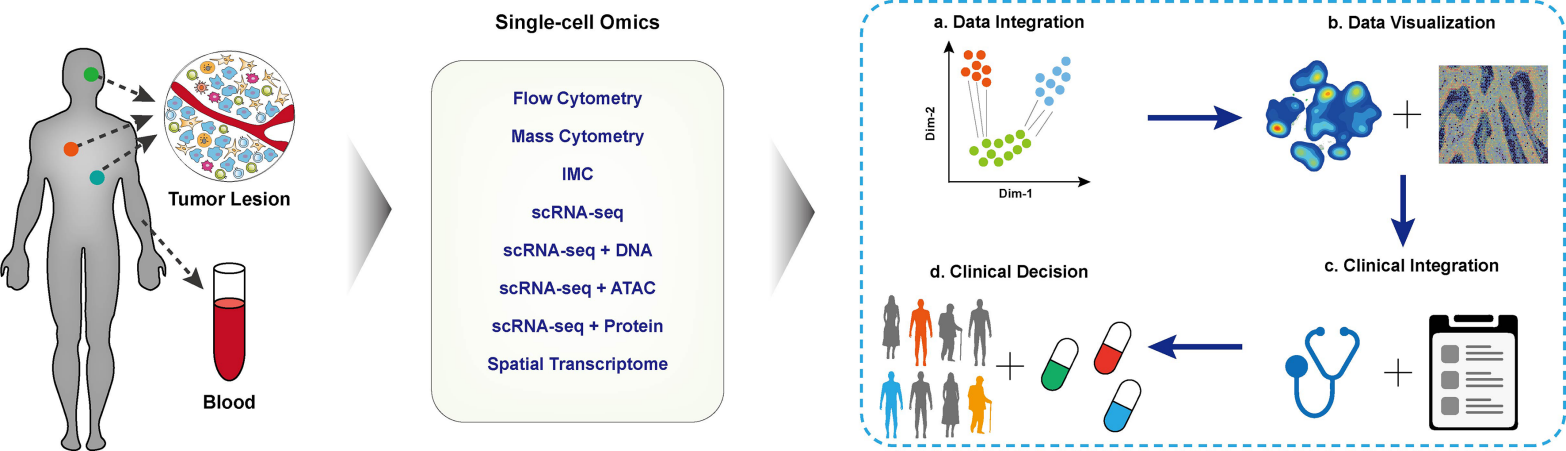
Applications of single-cell techniques in clinical cancer research. The schematic diagram of cancer research with single-cell techniques, blood or tissue samples of the designated patient cohort was collected and performed single-cell profiling. The collected data were integrated for downstream analysis and visualization. With in-depth integration with clinical characteristics, biomarkers for clinical decisions, disease prognosis, and tumor immunotherapy.

With the aid of single-cell methods, the heterogeneity of tumor cells and their interaction in the local microenvironment have been deeply and comprehensively interrogated. The single-cell data has been extensively used for identifying biomarkers for cancer diagnosis, prognosis prediction, and new treatable targets in designated clinical cohorts. The Human Tumor Atlas Network (HTAN) project (73) has put forward a framework of mapping tumor atlases in molecular, cellular, anatomical, and clinical fields, aiming to interrogate the single-cell data for clinical transitions thoroughly. In the following session, we mainly focus on applying different single-cell multi-omics techniques in establishing the cellular atlas of tumor ecosystem, T cell dynamics, and their interactions contributing to tumor diagnosis, treatment, and prognosis. The typical applications are correspondingly listed (**Figure 2** and **Table 1**).

**Table 1:** Selected cancer research with Single-Cell omic technologies.

Cancer type	Single-cell methods	Highlights	Ref
**Early lung adenocarcinoma**	CyTOF, scRNA-seq	Comparing the paired immune signatures across tumor lesion, normal lung tissue, and blood, Lavin et al. Identified the tumor lesion-specific immune regulations, especially the modifications of innate immune cells	(74)
**Breast cancer**	Imaging mass cytometry	The high-dimensional pathology images of breast cancers characterized the disease-related spatial resolved cellular signatures.	(77)
**Hepatocellular carcinoma**	scRNA-seq	In-depth integration of single-cell data with bioinformatic methods, Zhang et al. identified the migration of immune cells, especially the LAMP3+ dendritic cells, potentially contributing to lymphocyte activation.	(87)
**Myeloproliferative neoplasms**	scRNA-seq + genotyping	Integrating the cellular mutation genotypes and transcriptomic data, Nam et al. revealed the upregulation of NF-κB and IRE1-XBP1 pathways in mutated cells. And the modifications of mutations in transcriptomic outputs.	(82)
**Mixed-phenotype acute leukemias**	scRNA-seq + protein, scATAC-seq	By comparing the transcriptomic and epigenetic blood development maps between healthy and MPAL patients, Granja et al. uncovered the patient-specific regulatory networks, such as the RUNX1 regulation of CD69 in tumor patients.	(47)
**Primary pancreatic tumors**	scRNA-seq, spatial transcriptomics	With intersection analyses of scRNA-seq data and spatial transcriptomic data, Moncada et al. revealed the interactions of different cells in tumor microenvironments, especially the colocalization of inflammatory fibroblasts and cancer cells.	(86)
**Basal or squamous cell carcinoma**	scRNA-seq + TCR	Comparisons between the tissue TCR repertoires before and after immunotherapies, Yost et al. uncovered the new entered T cell clonotypes rather than the exhausted T cell clonotypes that may respond to immunotherapy.	(105)
**Hepatocellular carcinoma**	scRNA-seq	By comparing the immune landscape between primary and early-relapse HCC patients, Sun et al. indicated the innate-like CD8 T cells might contribute to an early relapse of HCC.	(119)
**Pancreatic ductal adenocarcinoma**	snRNA-seq, spatial transcriptomics	Comparisons of the PDAC samples before and after chemoradiotherapy, Hwang et al. revealed the basal rather than the classical phenotype of malignant cells might benefit the therapy efficiency with single-cell and spatial transcriptomic inspections.	(121)

### Dissecting Tumor Microenvironments at the Single-Cell Level

Taking advantage of high throughput and high dimensional proteomic single-cell analysis, CyTOF has been used to dissect the immune composition of TME in different types of tumors. In the study of early lung adenocarcinoma (74), Lavin et al. profiled the immune atlas in paired tumor lesions, normal lung tissues, and peripheral blood. They revealed a tumor-specific depletion of CD8^+^ T effector cells and the tumor-enriched macrophages with the expression of PPARγ potentially contributing to immune suppression in TME. This study provides potential immunotherapies for targeting macrophages in lung cancer. By comparing the immune atlas of clear cell renal cell carcinoma (ccRCC) and normal renal tissues (82), Chevrier et al. identified the polymorphic expressions of exhausted markers and CD38 on PD-1^+^ exhausted T cells in tumors and a special subset of CD38^+^ tumor-associated macrophages (TAM) highly associated with the immunosuppressed T cell subsets. Further integrating the tumor-infiltrating frequencies of immune cell subsets with clinical outcomes, they identified the abundance of several TAM subsets that can predict the progression-free survival of patients. Additionally, CyTOF and imaging CyTOF have also been combined with profiling the ecosystem of malignant cells and immune cells in breast cancer. Wagner et al. simultaneously compared the immune and malignant cell components of breast tumor, juxta-tumor, and mammoplasty tissue samples. The phenotypic abnormality of tumor cells and dynamics of immune cells suggests the tumor-immune combined phenotypes of breast tumor patients independent of the clinical grade and subtypes, suggesting the local interactions could be more critical for prognosis treatment efficacy (83). Using imaging CyTOF, Jackson et al. established the highly multiplexed molecular spatial maps of breast tumor microenvironments with different clinical subtypes and grades. Interrogating the single-cell pathology features, they further proposed that the subgroups of patients by pathology features in the tumor microenvironment could better predict patients’ overall survival and provide new strategies for clinical subtyping (75).

With higher dimension capability, scRNA-seq provides an opportunity to more broadly and systematically profile TME and its associated immune atlas with many more critical functional aspects (84). For example, with scRNA-seq, Azizi et al. identified the continuous cellular states of T cells and myeloid cells in breast cancers (85). They proposed that both TCR signals and environmental stimuli could module T cell functionality to combine TCR clonotypes with T cell phenotypes. scRNA-seq also enables more comprehensive lineage analyses to reflect the dynamic immune cell responses during tumorigenesis. With RNA Velocity analysis (64) and mitochondrial-based lineage tracing (86), Zhang et al. revealed that a subset of LAMP3^+^ dendritic cells could migrate from tumors to hepatic lymph nodes to trigger systematic adaptive immune responses (76).

Integrating the genotyping and single-cell transcriptomic data of myeloproliferative neoplasm cells, Nam et al. comprehensively delineated the contributions of CALR mutation in the differentiation of hematopoietic stem and progenitor cells (HSPCs). They also revealed that the CALR mutation more affected the cellular gene expressions at a later differentiation stage and further identified the mutation-specific activation of the IRE1-XBP1 pathway in HSPCs as a potential therapeutic target (77). With single-cell sequencing the genomics and transcriptomics of acute myeloid leukemia (AML) malignant cells, van Galen et al. identified six subsets of malignant AML cells across developmental hierarchies. They revealed the determination role of genotype in the compositions of AML cells in patients and further determined that the differentiated AML cells could suppress the function of T cells. The genotype-specific phenotype of AML cells and the immunosuppressive functionality of differentiated AML cells could further guide the genotype-specific immunotherapies in AML (87). Single-cell triple omics sequencing (scTrio-seq), a platform that simultaneously profiles genomic, epigenomic, and transcriptomic on individual cells, is able to delineate the more complex insights of the coordinated regulations of copy number variations, DNA methylation, and gene expressions in malignant cells of hepatocellular carcinomas and colorectal cancer (88, 89). Moreover, comparing the epigenomic regulatory networks of bone marrow and peripheral blood mononuclear cells between healthy and mixed-phenotype acute leukemia (MPAL) patients, Granja et al. uncovered the common regulation factors and revealed RUNX1 as an oncogene to upregulate CD69 in MPAL (47). Integrating scRNA-seq and spatial transcriptomic data in pancreatic ductal adenocarcinoma, Moncada et al. intersected the region-specific gene expression with cell type-specific gene expression. They revealed that the stress-response cancer cells were colocalized with IL-6 releasing inflammatory fibroblasts, supporting the IL-6 induced stress-response mechanism in cancers (78). Thus, the integration of single-cell multi-omics allows a more comprehensive exploration of cancer evolution, local cellular interactions, and immune regulations in the tumor microenvironment, strengthening our understanding of cancer pathogenesis and immune suppression (90, 91).

### Evolution of Cancer Cells in Tumorigenesis and Drug Resistance

In-depth single-cell characterization of cancer cells in the TME, and their dynamic regulations in tumorigenesis, metastasis, and drug responses can uncover the heterogeneity of cancer cells and their causality with clinical outcomes (90, 92). The scRNA-seq profiling of diverse cancer cells in oligodendroglioma patients revealed a subset of undifferentiated malignant cells with stem cell phenotypes and proliferating potentials, suggesting the primary roles of cancer stem cells (CSCs) in cancer evolution (93). Integrating the genetically engineered mouse models (GEMMs) and scRNA-seq, Marjanovic et al. mimicked and profiled the progression of human lung adenoma and adenocarcinoma. They identified a subset of TIGIT^+^ cells with the high-plasticity cell state (HPCS) and annotated these cells as transitioning tumor cells that contributed to tumor progression and chemoresistance (94). Neftel et al. also characterized four malignant cell subsets of glioblastoma using scRNA-seq with specific molecular features, and that the cellular transitions demonstrated the plasticity of malignant cells across distinct malignant cell subsets with additional cell barcoding and lineage tracing (95). All of these findings highlighted the impact of high-resolution single-cell profiling in understanding tumorigenesis and the evolution of cancer cells.

Metastasis is the dominant cause of the deaths of cancer patients, and its process is stochastic and dynamic (96). scRNA-seq study in human metastatic lung adenocarcinoma (LUAD) revealed a subset of cancer cells with distinct differentiation trajectory and gene signature of aggressive cell movement, proliferation, and apoptosis. And the gene signature of this cancer cell subset is enriched in later and metastatic tumor tissues and associated with a worse prognosis (97). Meanwhile, the applications of scRNA-seq and Cas9-enabled high-resolution lineage tracing of the xenograft model of LUAD cell line delineated comprehensive disseminate routes of metastatic cancer cells. And combining the phenotypical inspection of scRNA-seq data, Quinn et al. uncovered the characteristics of cancer cells with different metastatic ability and quantified their specific transcriptomic regulation in modulating metastasis (98).

The drug resistance of cancer cells severely limits the efficacy of chemotherapy or molecularly targeted therapies, and the cellular states and responses during treatments can determine further disease progression (4). In breast cancer, the single-cell profiling of docetaxel-resistant MCF7 breast cancer cells revealed a subset of cells with a stem-like phenotype and identified LEF1 as the critical molecule regulator in drug resistance (99). In melanoma, an immune evasion-specific malignant cell program identified by scRNA-seq can predict the clinical responses of immune checkpoint inhibitors (ICIs). Targeting the signal activation of CDK4/6 in this program can repress the drug resistance program and enhance the ICI efficacy (100). Meanwhile, a multimodal method (Perturb-CITE-seq) was applied to characterize the mechanisms of resistance of ICIs. Integrating the simultaneously RNA and protein profiling with Cas9 genomic knockout screens, Frangieh et al. validated the known mechanisms of resistance to ICIs, and further revealed a novel CD58 related resistance mechanism. Specifically, they found that downregulating the expression of CD58 could induce the expression of PD-L1 on malignant cells and reduce the co-stimulatory signal of the CD58-CD2 axis on CD8^+^ T cells (101). Overall, the comprehensive interrogating of the cellular responses and drug resistances in malignant cells could uncover the new treatable targets and guide the combined therapies for cancer treatments.

### T Cell Responses and TCR Repertoire in Tumor Immunity

T cells are essential adaptive immune cells that mediate tumor immunity. The promising immune checkpoint blockade (ICB) therapies mainly target T cells and recover T cell immunity through disrupting PD-1/PD-L1 and CTLA-4/CD80 or CD86 interactions or specifically activating tumor-antigen-specific T cell clones (102, 103). Unfortunately, only a small part of patients has beneficial responses with recovered anti-tumor T cell responses. Improving the ICB efficacy requires a more comprehensive understanding of dynamical T cell responses in patients during tumorigenesis and ICB treatments (104).

Platforms that integrate scRNA-seq data and scTCR-seq in individual T cells, such as Smart-seq3 and 10X Genomics single-cell immune profiling, enable a more precise delineation of immune responses and lineage tracking of T cells in tumorigenesis or under immunotherapy treatments (105). Smart-seq3, a representative of full-length sequencing platform, could read full-length CDR3 sequences of TCRαβ chains in single cells but with limited throughput (23). 10X single-cell immune profiling, a commercial droplet-based platform that integrates TCR enrichment procedures, enables more efficient immune profiling of T cells (68).

Every T cell owns a unique TCR, which provides a valuable lineage tracking marker to investigate the dynamics of T cells, including T cell clonal expansion, functional changes of a TCR clonotype, and T cell migration across different tissues. The T cell landscape with the information of paired TCR α and β chains in liver cancers comprehensively discloses the transition route of exhausted CD8^+^ T cells in HCC and highlights that a subset of CD8^+^ T cells with intermediate levels of PDCD1 and TIGIT can be the target cells for immunotherapies (106). In another work, Zhang et al. developed an analysis algorithm (STRATRAC) to quantify the T cell expansion, migration, and transition with paired TCR repertoires (107). With the T cell transition analysis of exhausted CD8^+^ T cells in colorectal tumors, Zhang et al. revealed a tight association of these cells with effector memory CD8^+^ T cells but independence of the development trajectory of effector memory and recently activated effector memory CD8^+^ T cells, suggesting a TCR-dependent fate decision in tumorigenesis. These works strengthen our understanding of the dynamics of T cell exhaustion in tumorigenesis. Furthermore, in-depth profiling of T cell dynamics before and after anti-PD-1 therapy in basal or squamous cell carcinoma suggests the newly entered T cell clonotypes, rather than the exhausted T cell clonotypes, respond to anti-PD-1 immunotherapy (79).

T cells are the dominant targets of immunotherapies, and their responses after immunotherapy treatments are critical to evaluate the clinical efficacy (108). Thereby, the clonal expansions and the accordant changes of TCR repertoires in tumors, normal adjacent tissue, and peripheral blood can be used for predicting the clinical responses to immunotherapies (109). Meanwhile, multiple computational methods have been developed to connect the similarities of TCR sequences with T cell functionalities, which would expand the applications of TCR repertoires in cancer research (110–112). Besides, the comprehensive inspection of T cells in tumors also directs adoptive T cell transfer (ACT) in cancer therapies to identify tumor-responsive T cells (104).

### The Molecular Biomarkers for Tumor Diagnosis and Prognosis

The heterogeneities of the tumor microenvironment and strikingly different clinical outcomes in tumor patients require comprehensive molecular profiling to guide the personalized therapies. Multiple initiatives have been founded to identify tumor-specific biomarkers to facilitate better clinical decisions using integrative single-cell omics data analyses (73, 113, 114).

Several groups focused on seeking potential disease or prognosis-related biomarkers using CyTOF. Comparing the peripheral immune atlas of 20 melanoma patients before and after anti-PD-1 immunotherapy, Krieg et al. found that the frequency of CD14^+^CD16^-^HLA-DR^+^ monocytes in peripheral blood before treatment was highly correlated to the response of anti-PD-1 immunotherapy and thus could help to stratify patients before anti-PD-1 immunotherapy treatment (115). In a similar study of dissecting immune profiling in classical Hodgkin lymphoma (116), the peripheral TCR diversities in CD4^+^ T cells at baseline and during PD-1 blockade therapy were related to the clinical responses. Meanwhile, comparing the development of B cells in B cell precursor acute lymphoblastic leukemia patients and healthy controls, Good et al. revealed that the abnormal expansions of specific B cell subsets during development could predict disease relapse at the time of diagnosis (117). Although implemented in a small patient cohort, all of these strongly suggest the predictive capability of cellular composition changes in prognosis prediction and disease monitoring. Besides, the spatial inspection by imaging CyTOF in molecular colocalization of metastatic melanoma highlighted the association between the prior expression of β2m in TME and clinical outcomes of immunotherapy (118). Profiling the subcellular molecular maps of 483 breast tumor samples using imaging CyTOF in the METABRIC cohort, Ali et al. uncovered the genomic regulation of local tumor ecosystems, including cellular compositions and cellular neighborhoods. They intensively examined their clinical predictive roles in the prognosis of breast cancer (119). All these studies demonstrate the power of the single-cell CyTOF system in finding potential molecular biomarkers for cancer prognosis and predicting treatment efficacy.

scRNA-seq data has also been used in seeking molecular and cellular basis of TME. The distinct transcriptional signatures of malignant cells with different genomic backgrounds help classify tumor subtypes and the design of targeted treatments in a higher resolution (120). Comparing the ecosystems of primary and early-relapse HCC tissues, Sun et al. indicated and validated the enrichment of innate-like CD161^+^CD8^+^ T cells with limited cytotoxic ability in relapsed HCC tissues and may have poor response to the subclonal neoantigens in early-relapsed tumor cells, providing new targets to restrain HCC relapse (80). With the single-cell inspection of tumor-infiltrating lymphocytes in breast cancers, Savas et al. revealed a gene signature of tissue-resident memory CD8^+^ T cells rather than the CD8 alone could better predict the patient’s survival, suggesting these cells are potential regulatory targets of immunotherapy in breast cancer (121). More recently, Hwang et al. delineated the molecular taxonomy changes of TME in pancreatic ductal adenocarcinoma patients treated with or without neoadjuvant chemotherapy and radiotherapy by using the integrated single-nucleus RNA sequencing and spatially resolved transcriptomics analyses (81). They found that the basal-like or classical-like reprogramming of malignant cells was associated with distinct immune infiltration in tumors and further affected the treatment outcomes and clinical decisions.

Despite the durable clinical responses of chimeric antigen receptor T cell (CAR-T) therapy in treating hematological malignancies, the response rate, adverse events, and neurotoxicity during CAR-T treatment can vary across patients (122, 123). Single-cell omics have been applied to uncover the molecular biomarkers of clinical responses and monitor CAR-T cells’ functional changes for better clinical application (124, 125). Using scRNA-seq, Deng et al. intensively interrogated the transcriptomic phenotypes of CAR-T cells in infusion products (IPs) with their consequent clinical outcomes on large B cell lymphoma patients (124). They revealed that the enrichment of the memory phenotype of CAR-T cells within IPs lead to positive clinical responses but that the enrichment of exhaustion phenotype of CAR-T cells associated with disease progression. Moreover, they also identified a subset of monocyte-like cells in IPs significantly related to high-grade immune effector cell-associated neurotoxicity syndrome (ICANS). Furthermore, Sheih et al. comprehensively profiled the temporal changes of CD8^+^ CAR-T cells within IPs, peripheral blood early after infusion and after the peak of CAR-T cell expansion (125). Using the paired scRNA-seq and scTCR-seq, they identified the CD8^+^ CAR-T cells, within timely increased relative frequency (IRF) clonotypes, highly expressed the gene signatures of T cell cytotoxicity and proliferation, suggesting their effective roles in anti-tumor responses. These studies guide the further applications of single-cell omics to deeper understand the mechanistic insights of effective CAR-T therapy, which would shed light on optimizing CAR-T therapy and uncovering the molecular biomarkers for predicting clinical outcomes.

Furthermore, a new concept of a three-dimensional cell atlas during tumor evolutions has been introduced by Human Tumor Atlas Network (HTAN) project (73), indicating the molecular, spatial, and clinical inspections of human tumors, which would help uncover the fundamental mechanisms of tumorigenesis and new biomarkers for cancer screening, tumor metastasis, cancer immunotherapy, and drug responses in the future.

## Perspectives

In this review, we comprehensively summarize the development of multiple single-cell omics techniques and their applications in cancer biology and cancer immunology. These innovative methods have extensively enhanced our understanding of tumorigenesis, the mechanisms of tumor-induced immune escape, and the dynamic responses to different tumor treatments. Although significant progress has been made, multiple challenges still exist, which could limit current studies and need to be further solved. In the CyTOF system, the preset and limited number of designated markers hinders the identification of novel or rare cell populations. Additional rare elements to increase the detectable number of channels are required to further assist their applications in the clinical field. In the scRNA-seq system, as the number of detected genes, the transcript-length coverage, and the measurement throughput varied across different platforms and assays, it is challenging to integrate and compare single-cell data from different systems. Meanwhile, the limited transcript capture efficiency of scRNA-seq methods leads to a high dropout of scRNA-seq data, resulting in a higher noise level than bulk RNA-seq (126). The common usage of 3’ end transcript capture in scRNA-seq methods involves many non-informative transcripts, making the specific examination of interested transcripts infeasible and wasting the sequencing cost (127). Thus, an optimized system that is able to economically and efficiently generate scRNA-seq data with high data quality and uniform data format is emergently desired to achieve robust analysis of larger sample cohorts. Meanwhile, a more prospective direction in the future is to profile single cells with integrated multi-omics to enable better and deeper profiling of the complicated tumor ecosystem. Moreover, new computational tools to improve the integrated data quality, facilitate the biological interpretation, and speed up the analysis procedures are valuable to be developed.

Single-cell data-driven clinical translation is important and promising in cancer diagnosis and treatment. Due to the expensive cost of single-cell methods, the enrolled patient cohort in current cancer research is very small, leading to inconsistent and non-repeatable biological findings. How to interrogate enormous single-cell features with clinical outcomes is computationally challenging and requires more external validations. Moreover, the tissue sites, sample status, isolation methods, and timepoint for sample resections can be varied across different clinical studies, leading to unstable and non-repeatable single-cell biomarkers found in the clinical field. Thus, a more feasible single-cell framework for performing large-scale clinical studies and the resources for sharing and exploiting the published single-cell data mainly in the cancer field, are urgently needed for better clinical translation in the future.

In summary, single-cell omics techniques will be indispensable for investigating both basic and clinical problems in tumor biology, tumor immunology, and tumor immunotherapy in the future, as they provide broader and deeper insights in large patient cohort to inspire more precise and personalized medicine in cancer treatments.

## Author Contributions

JL, WY, WC, and SQ wrote the manuscript. TZ helped with the preparation of the figure, and all authors provided thoughtful advice to revise the manuscript. All authors contributed to the article and approved the submitted version.

## Funding

This project was supported by the National Natural Science Foundation of China 31600751 to WY and the Ministry of Science and Technology of China No.2017ZX10203205 to WC.

## Conflict of Interest

HS and WY are both co-founders of, and WC is the scientific consultant of Zhejiang Puluoting Health Technology Co., Ltd (PLT). HS is the CEO of the PLT.

The remaining authors declare that the research was conducted in the absence of any commercial or financial relationships that could be construed as a potential conflict of interest.
